# How to Synthesise High Purity, Crystalline d‐Glucaric Acid Selectively

**DOI:** 10.1002/ejoc.201701343

**Published:** 2017-12-06

**Authors:** Robert D. Armstrong, Benson M. Kariuki, David W. Knight, Graham J. Hutchings

**Affiliations:** ^1^ Cardiff Catalysis Institute School of Chemistry Cardiff University Park Place CF10 1AQ Cardiff UK

**Keywords:** Carbohydrates, Glucaric acid, Platform molecule, Chemoselectivity

## Abstract

Glucaric acid has potential applications in food, pharmaceutical and polymer industries yet no methodology exists within the public domain for isolation of this key bio‐derived platform molecule as a pure, crystalline solid. Here we demonstrate the difficulties, which arise in doing so and report development of a process for derivation of free‐glucaric acid from its Ca^2+^/K^+^ glucarate salts, which are both commercially available. Employing Amberlyst‐15 (H^+^) exchange resin and azeotrope drying, powdered glucaric acid is prepared at > 99.96 % purity in 98.7 % dry yield.

## Introduction

Feedstock homogeneity is a key concern within the chemical industry. This is particularly true for bio‐derived product streams, which often lack the purity offered by refined‐fossil fuel sources. Glucaric acid is a C_6_ aldaric acid which is produced commercially through the oxidation of glucose with nitric acid. Isolated as its monopotassium salt at *ca*. 40 % yield with oxalic, tartaric and 5‐ketogluconic acids as by‐products, this process dates back to 1888 and meets a majority of the ever‐increasing global demand for glucaric acid derivatives.^1^, ^2^, ^3^ Recently, glucaric acid has garnered worldwide interest due to its many roles in the food supplement, pharmaceutical and polymer industries,^4^, ^5^, ^6^, ^7^, ^8^, ^9^, ^10^ the latter being as a bio‐derived precursor to Nylon 6,6. Indeed, the US Department of Energy recently recognised it as a key sugar derived platform molecule.^11^ Meanwhile, studies have shown this aldaric acid (administered as a calcium salt) and its derivatives to be effective inhibitors of β‐glucuronidase and that this effects a reduction in covalent bonding of procarinogenic metabolites to DNA.^9^, ^12^ Recently companies such as Rennovia Inc. have commercialised catalytic processes for the preparation of glucaric acid from glucose, eschewing the nitric acid oxidant in favour of a platinum‐catalysed aerobic oxidation cycle.^4^, ^5^, ^13^ This is in line with a recent trend towards catalytic oxidation of glucose to glucaric acid.^14^, ^15^, ^16^, ^17^, ^18^, ^19^, ^20^, ^21^ However despite this, K and Ca glucarate salts remain the commercially available forms of the acid.

**Scheme 1:**
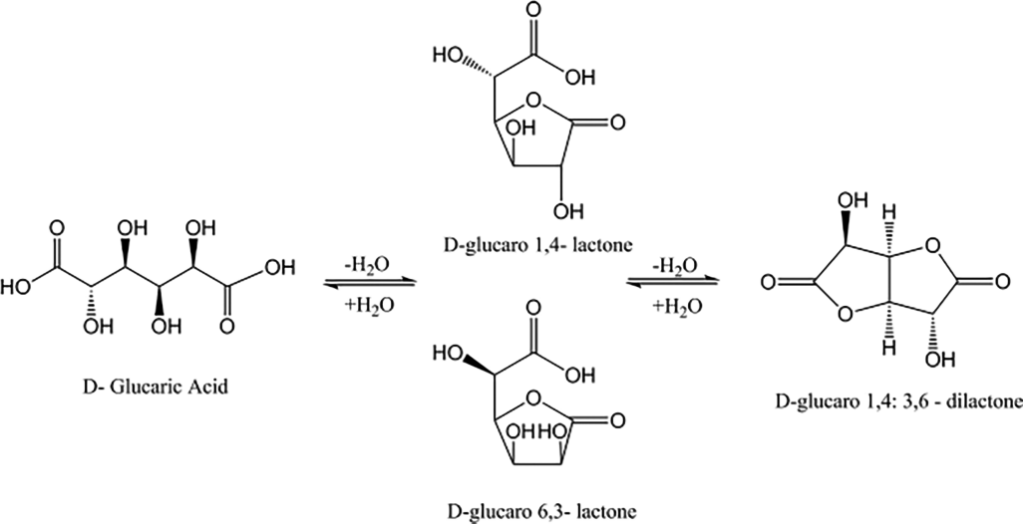
Equilibration of d‐glucaric acid and its mono and dilactone derivatives.

Although the patent literature alludes to processes for deriving the free diacid from its salts,^4^, ^22^ no study has as yet confirmed this through combined elemental/spectroscopic analysis with accompanying dry yields. As glucaric acid is known to exist in equilibrium with the lactones; d‐glucaro‐1,4‐lactone and d‐glucaro‐6,3‐lactone, and the dilactone d‐glucaro‐1,4:6,3‐dilactone^23^ (Scheme 1), a method for the preparation of high purity isolated glucaric acid is of broad interest. Three instances whereby the preparation of glucaric acid from glucarate salts is claimed have been reported.^4^, ^22^, ^24^ Kiely et al. detailed a process whereby an aqueous solution of monopotassium glucarate was ion exchanged with Dowex 50WX8 (H^+^) ion exchange resin to yield a solution of d‐glucaric acid, which was filtered and freeze dried to yield a syrup, claimed to comprise 100 % of the free diacid.^22^ The same authors later reported another route, utilising the same ion exchange step followed by lyophilisation, with glucaric acid subsequently recovered through seed assisted crystallisation, overnight at 4 °C.^24^ Following trituration with acetone, glucaric acid was recovered, though no yield was reported.^24^ Meanwhile Boussie et al. reported the preparation of glucaric acid from calcium glucarate, though provided no methodology and went on to acknowledge the presence of glucaro lactones and dilactones in addition to the target aldaric acid.^4^ While these results are promising, the lack of experimental detail and analysis of the derived acid means that a proven, reproducible methodology for the preparation of solid d‐glucaric acid of appreciable purity and yield is still needed. ^1^H and ^13^C chemical shifts have been previously reported for the glucarate derivatives shown in Scheme 1.^23^

## Results and Discussion

**Figure 1:**
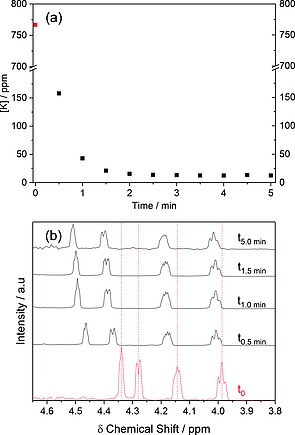
ICP‐MS (a) and ^1^H NMR analyses (b) showing temporal conversion of K‐glucarate to d‐glucaric acid through ion exchange of K^+^.

Addition of Amberlyst‐15 (H^+^) ion exchange resin (5.0 g) to an aqueous solution of K‐glucarate (100 mL, 0.02 m, 0.5 g, 2 mmol, *Sigma Aldrich*) led to > 99 % exchange of K^+^ for H^+^ after just 5 min (Figure 1a). ICP‐MS analysis showed a decrease in potassium concentration from 767.8 ppm to 12.6 ppm over this period. Containing four stereocentres, glucaric acid presents four chemically inequivalent ^1^H resonances in D_2_O at *δ* = 4.00, 4.17, 4.39 and 4.50 ppm (Figure 1b. d‐Glucaric acid protons c, b, d and a respectively).^23^

**Figure 2:**
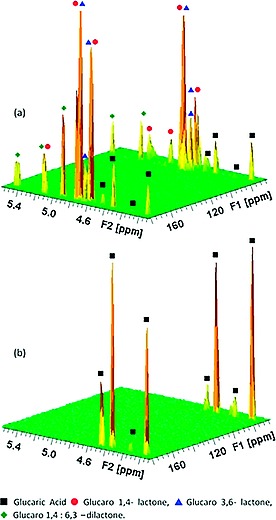
3D contour plots showing HMBC nmr spectra of products of K‐glucarate ion exchange recovered from H_2_O (a) and a 5:95 H_2_O/MeCN azeotrope (b).

**Figure 3:**
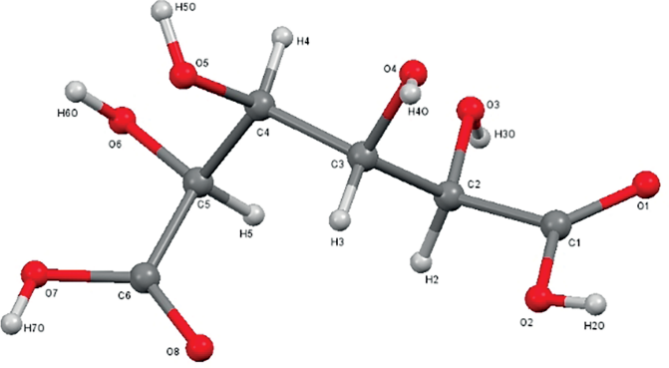
The X‐ray crystal structure of as‐prepared d‐glucaric acid.

No significant resonances attributable to glucaro‐lactones or dilactone were observed (Figure 1b) at room temperature (D_2_O). A downfield shift of resonances H_a_ and H_d_ upon exchange of K^+^ with H^+^ is consistent with lessened shielding of the carbonyl‐adjacent protons. Removal of the solvent (H_2_O) by rotary evaporation (50 mbar, 50 °C) yielded an orange syrup, consistent with previous studies.^13^ The observed inability to fully dry this mixture under these conditions is due to hydrogen bonding between the highly functionalised C_6_ derivatives and water. HMBC NMR analysis of this syrup dissolved in D_2_O showed numerous ^13^C–^1^H interactions (Figure 2a). These correlate strongly with reference values for 2–4 bond H‐C coupling in glucaric acid as well as its lactone and dilactone derivatives.^23^ Indeed, the mixture was determined by ^1^H NMR to comprise of glucaric acid (11 %), glucaro 1,4 lactone (12 %), glucaro 6,3‐lactone (41 %) and glucaro 1,4:6,3 dilactone (36 %). As it had previously been proposed that freeze drying of this syrup yields glucaric acid, this was assessed through freezing in liquid N_2_ followed by evacuation at 50 °C, with HMBC analysis of the resulting thick orange syrup indicating that further lactonisation (ESI Figure S1) had occurred.^22^ With an aim to decrease the temperature required for solvent removal, and thereby to minimise lactone formation, the solubility of K‐glucarate in both polar protic and polar aprotic organic solvents was assessed (ESI Figure S2). The K‐glucarate salt was found to be insoluble in organic solvents. This meant that the ion exchange step can only proceed in the presence of water. To significantly lower the temperature required for in vacuo water removal, a water‐containing azeotrope was sought. Acetonitrile forms azeotropes with water.^25^ Binary solvents comprising acetonitrile and water were therefore assessed (ESI Figure S3). At high acetonitrile volume fractions, K‐glucarate solubility was found to be low, indeed a MeCN/H_2_O azeotrope (5.8 wt.‐% H_2_O)^25^ showed *ca*. 0.31 mm solubility of the salt, far lower than observed for H_2_O. To circumvent solubility limitations, a two‐step approach was thus taken, comprising dissolution of K‐glucarate (0.02 m, 0.5 g, 2 mmol) in water (100 mL) followed by ion exchange with Amberlyst‐15 (H^+^) (5.0 g, 5 min). The solution was recovered by filtration (Amberlyst washed with H_2_O, 10 mL) and then azeotroped with acetonitrile (1.9 L).^25^ The solvent was then recovered by rotary evaporation (50 mbar, 22 °C) to yield an off‐white solid. This was dissolved in D_2_O and analysed by HMBC NMR (Figure 2b). All C–H interactions were attributable to d‐glucaric acid (2–4 bond interactions), with no evidence of lactone or dilactone derivatives. Elemental analysis showed the white solid to contain 0.09 wt.‐% K, equating to > 99.4 % exchange of K^+^ (ESI Figure S4). Furthermore it was determined that a second exchange with Amberlyst (5.0 g, 5 min)(10 mL of H_2_O washing) effected further exchange, yielding solid d‐glucaric acid (98.7 % dry yield) containing 0.006 wt.‐% K (δ =60 ppm by dry mass, 99.96 % K^+^ exchange)(ESI Figure S5). Melting point 111 °C (ref. 125.5 °C *PhysProp* 26). IR ν̃ = (C=O stretches) = 1724.0, 1687.3 cm^–1^ and ν̃ = (O–H stretches) = 3484.7 cm^–1^, 3286.0 cm^–1^, 3158.7 cm^–1^, (ESI Figure S6) ^1^H *δ* = 4.00, 4.17, 4.39 and 4.50 ppm (ESI Figure S7, 600 mHz, D_2_O). CHN analysis (*Warwick Analytical Service*) C 34.3 %wt/wt, H 4.8 %wt/wt (Formula C_6_H_10_O_8_ = C 34.3, H 4.8 %). Without further purification or separation, a crystal representative of the bulk dried product was analysed by single‐crystal X‐ray diffraction (Figure 3; Supporting Information), d‐glucaric acid adopts a bent conformation.^24^

**Figure 4:**
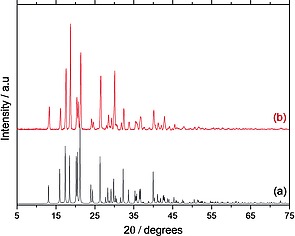
The recorded X‐ray powder diffraction pattern (a) and predicted pattern based on single‐crystal X‐ray diffraction indices (b) of d‐glucaric acid.

Furthermore, the powder X‐ray diffraction pattern of d‐glucaric acid is reported for the first time (Figure 4a). The diffractogram (Figure 4a) is consistent with that predicted from crystallographic indices derived through single‐crystal X‐ray diffraction (Figure 4b), suggesting that the structure in Figure 3 is representative of the bulk sample. When dissolved in D_4_ acetic acid, no H_2_O ^1^H resonances were observed, suggesting complete removal of the aqueous phase (ESI Figure S8). When utilising the other commercially available glucarate salt (Ca‐glucarate) which shows lower solubility in water (0.002 m, 0.06 wt.‐%, STP), recovered ion exchange products from H_2_O and MeCN: azeotrope solvents showed comparable compositions to those derived from K‐glucarate. Indeed the HMBC spectrum of the solid recovered through azeotropic drying was consistent with d‐glucaric acid (100 %) (ESI Figure S9). When dried from H_2_O alone (50 mbar, 50 °C), lactone and dilactone formation was observed, consistent with K‐glucarate studies (ESI Figure S10).

A preliminary optimisation of this process was carried out. To this end the solubility of K‐glucarate in H_2_O was determined (0.07 m, 1.7 wt.‐%, STP). This translated to a 250 % increase in volume‐yield of d‐glucaric acid (increasing from the original 0.02 m to 0.07 m), with NMR purity maintained as in Figure 2b. Additionally, to facilitate the azeotrope evaporation stage, the effect of bath temperature on the purity of recovered d‐glucaric acid was assessed (ESI Figure S11 note: the strong solvent peak for residual H_2_O from D_2_O has been removed for clarity). With increasing bath temperature, an increasing degree of lactonisation was observed by ^1^H NMR spectroscopy. It is clear therefore that low temperature solvent removal (< 28 °C) is vital in preparing high purity d‐glucaric acid through ion exchange.

## Conclusion

In summary, we present the first reported route to deriving the platform molecule d‐glucaric acid (> 99.96 % purity), as an isolated crystalline solid at high yield (> 98 %) from the commercially available glucarate; K^+^ and Ca^2+^ salts. In line with previous studies, an ion exchange resin is utilised in forming an aqueous solution of the free acid. It is shown through 2D HMBC NMR studies that previously reported methods for product recovery, which require relatively high temperatures for water removal, then lead to significant dehydration/equilibration to form lactone and dilactone glucarate derivatives. In contrast, the reported synthesis allows for the selective isolation of the diacid as a dry powder through azeotroping an aqueous solution of d‐glucaric acid with acetonitrile, which allows for low temperature solvent removal. This study paves the way to d‐glucaric acid being used by researchers in the chemical, food, pharmaceutical and polymer industries, where studies are currently restricted to pre‐ion exchanged aqueous solutions or K^+^/Ca^2+^ glucarate salts.

## Experimental Section

NMR spectra were collected on a Bruker Avance 500 MHz (11.7 Tesla; multinuclear) NMR spectrometer. Spectra were normalised to TMS (^1^H δ** = **0.0 ppm). Powder X‐ray diffraction was performed using a PANalytical X'PertPRO X‐ray diffractometer, with a CuΚα radiation source (40 kV and 40 mA) and Ni attenuator. Single‐crystal XRD data were collected on an Agilent SuperNova Dual Atlas diffractometer. CCDC 798054 (for glucaric acid) contains the supplementary crystallographic data for this paper. These data can be obtained free of charge from The Cambridge Crystallographic Data Centre.

## Supporting information

Supporting Information
