# Supplementary material for: How to Synthesise High Purity, Crystalline d‐Glucaric Acid Selectively
Source: European J Org Chem. 2017 Dec 6;2017(45):6811–4. doi: 10.1002/ejoc.201701343 (PMC5861666; doi:10.1002/ejoc.201701343)
Supplement: Supplementary file 1 — Supporting Information [file EJOC-2017-6811-s001.pdf]

Eur. J. Org. Chem. **2017** · ISSN 1099–0690

https://doi.org/10.1002/ejoc.201701343

SUPPORTING INFORMATION

Title: How to Synthesise High Purity, Crystalline D-Glucaric Acid Selectively
Author(s): Robert D. Armstrong,* Benson Kariuki, David W. Knight, Graham J. Hutchings*

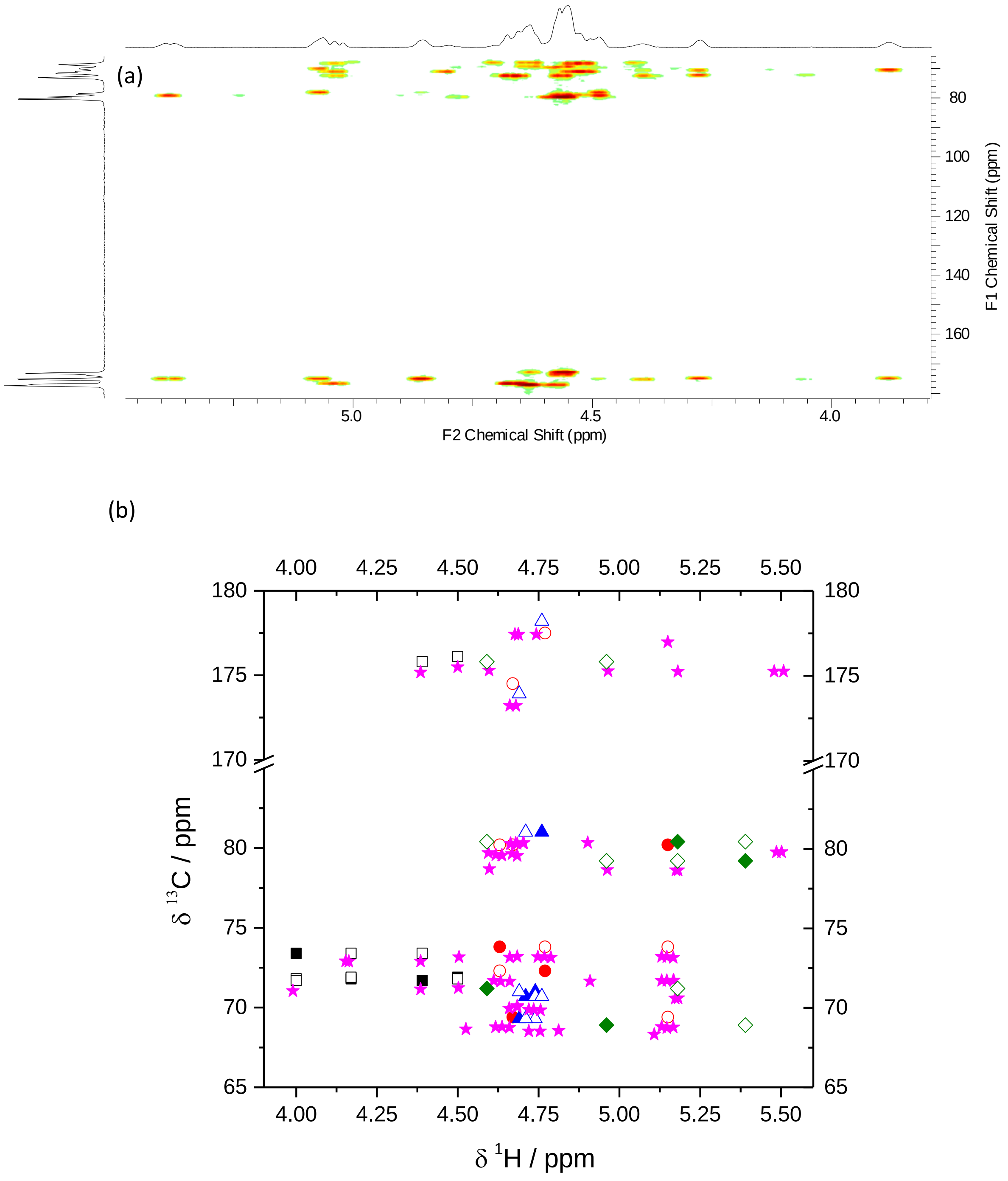

Fig. S1 HMBC spectrum (a) and spectral assignment (b) of K- glucarate exchange product recovered from H_2O through rotavap drying (50 mbar, 50 °C) followed by freeze drying.

■□ Glucaric Acid ●○ Glucaro 1,4- lactone, ▲△ Glucaro 3,6- lactone, ◆◇ Glucaro 1,4 : 6,3 – dilactone. Filled and empty symbols represent expected 1 and 2 bond ^{13}C – ^{1}H interactions respectively. ★Isolated product.

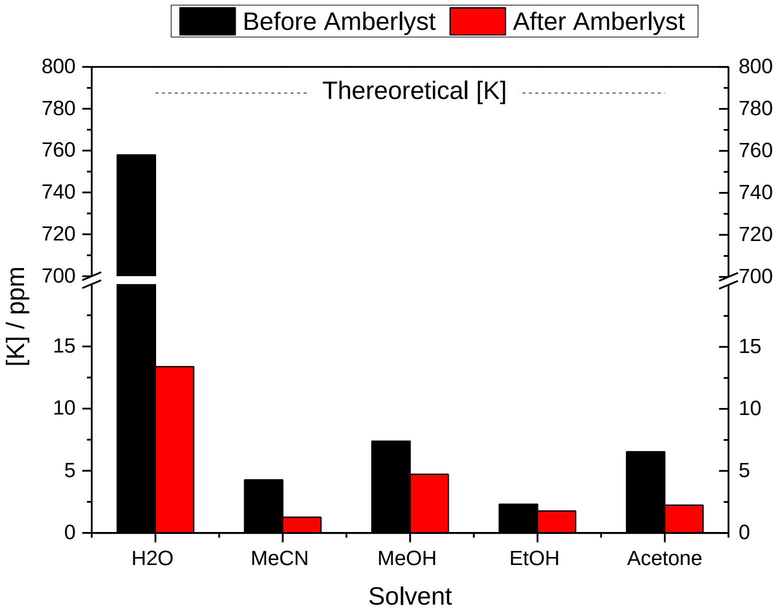

Fig. S2 Determination of the solubility of K-glucarate in polar solvents. Conditions; K-glucarate (0.05 g), Solvent (10 ml), room temperature, vigorous stirring. Where applicable Amberlyst-15 (0.5 g).

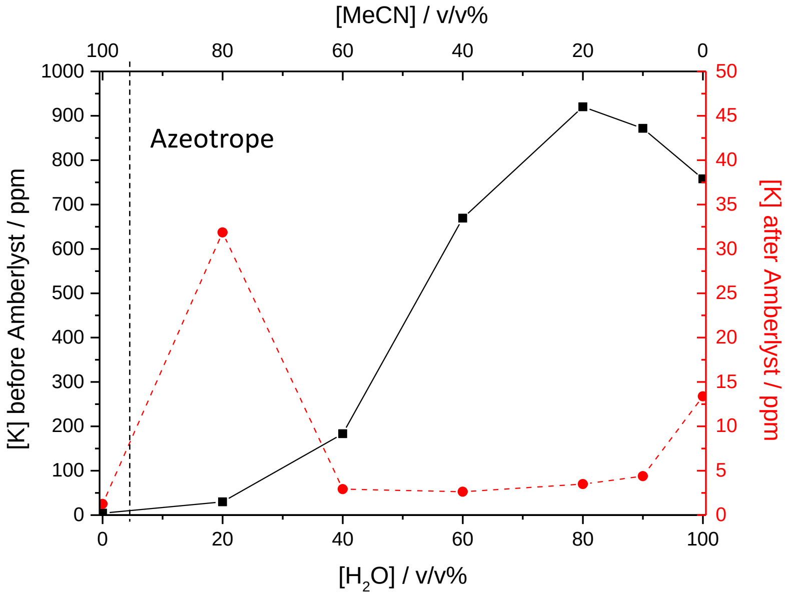

Fig. S3 Determination of the solubility of K-glucarate and efficacy of K^+ exchange in binary H_2O/ MeCN solvents of differing composition.

Conditions; K-glucarate (0.05 g), V_{total} solvent (10 ml), Stirred 1 h. Sampled (filtered sample) followed by AES. Then add Amberlyst (0.5 g) and stir for 5 min. Filter and AES.

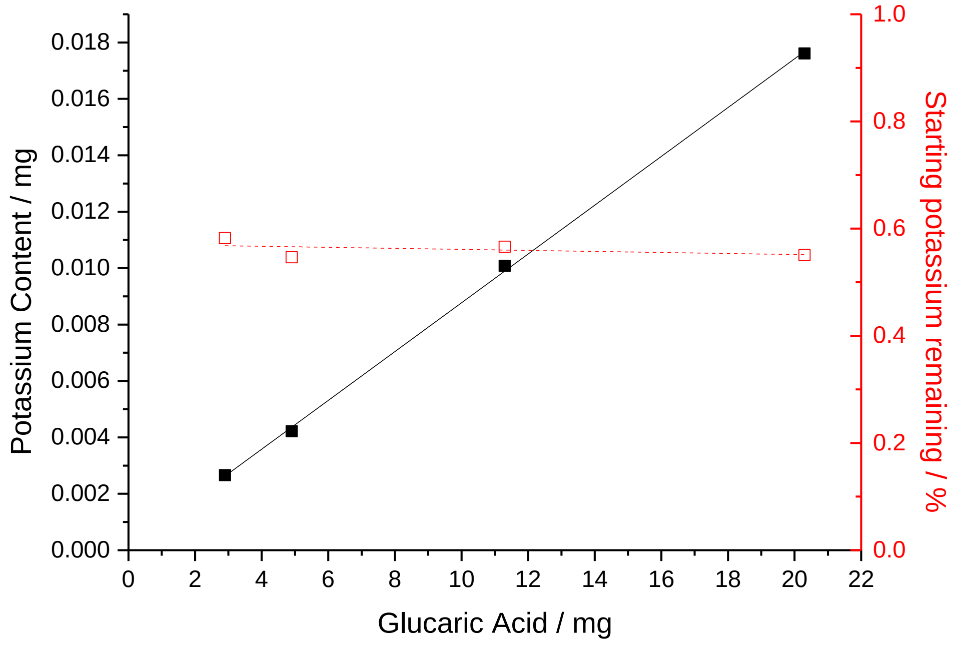

Fig. S4 Elemental analysis of azeotrope- dried glucaric acid.

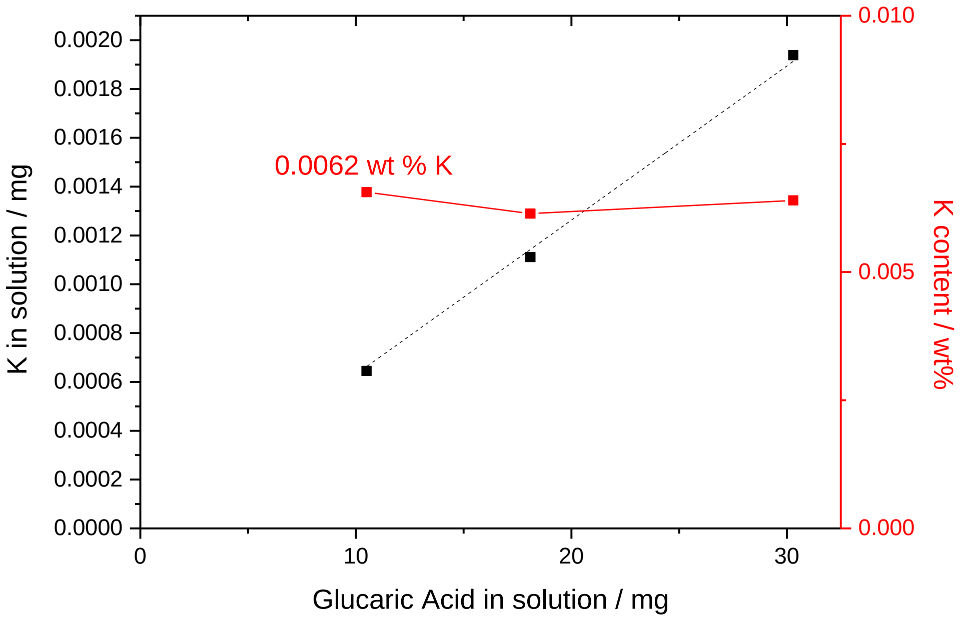

Fig. S5 Elemental analysis of azeotrope- dried glucaric acid following 2 consecutive H^+ exchanges.

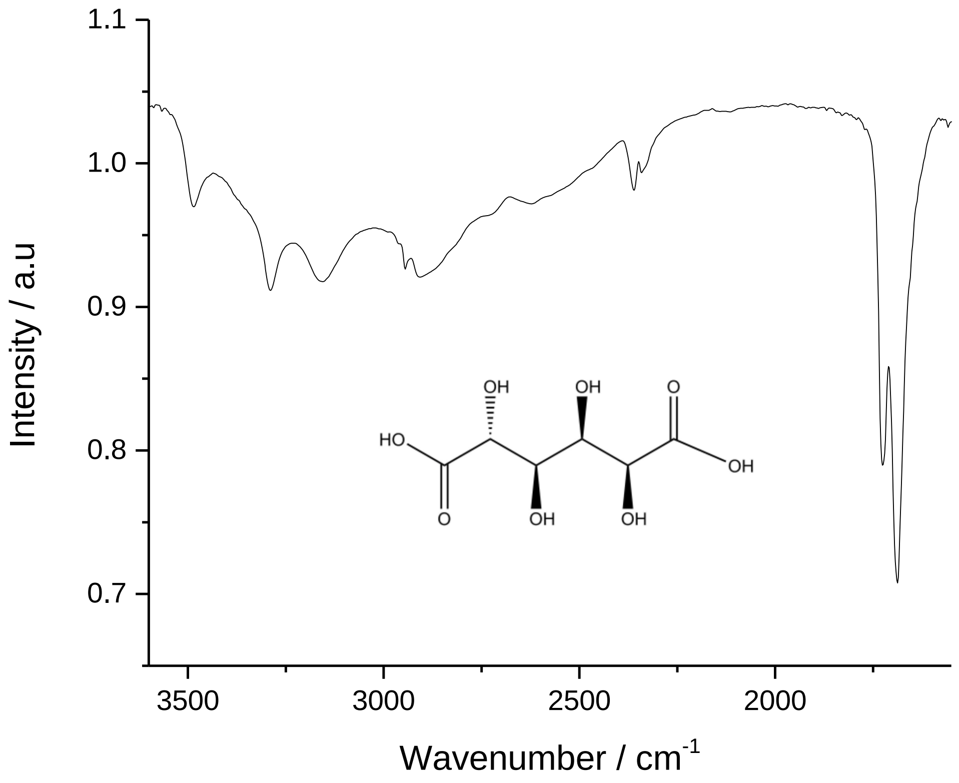

Fig. S6 IR spectrum of crystallised glucaric acid

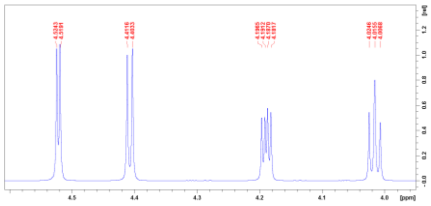

Fig. S7 NMR spectrum of synthesised glucaric acid collected at 600 mHz in D_2O

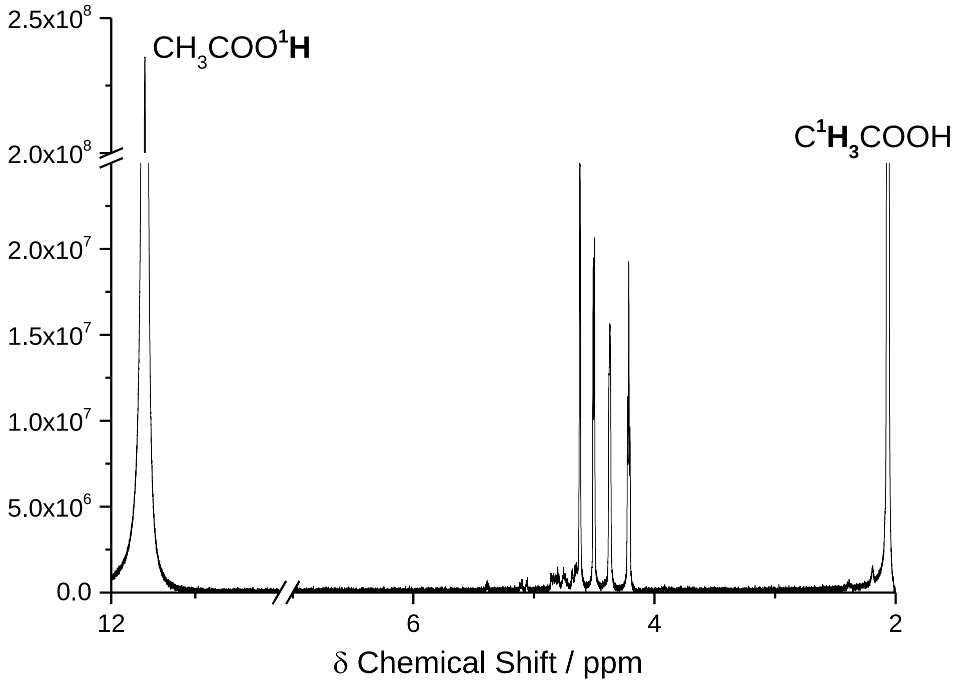

Fig. S8 ^{1}H NMR spectrum of azeotrope- dried Glucaric Acid dissolved in D_4 acetic acid, showing no H_2O resonances.

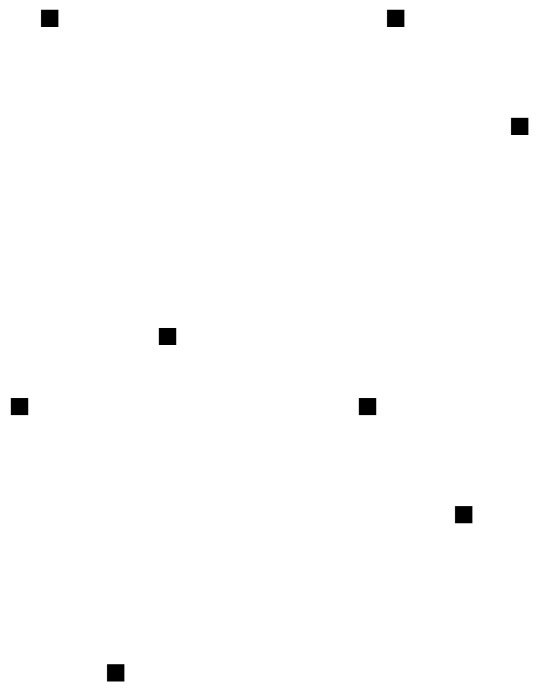

Fig. S9 HMBC spectrum of Ca- glucarate exchange product recovered from H_2O/ MeCN azeotrope through rotavap drying (50 mbar, 22 ^{o}C). ■ D-Glucaric Acid

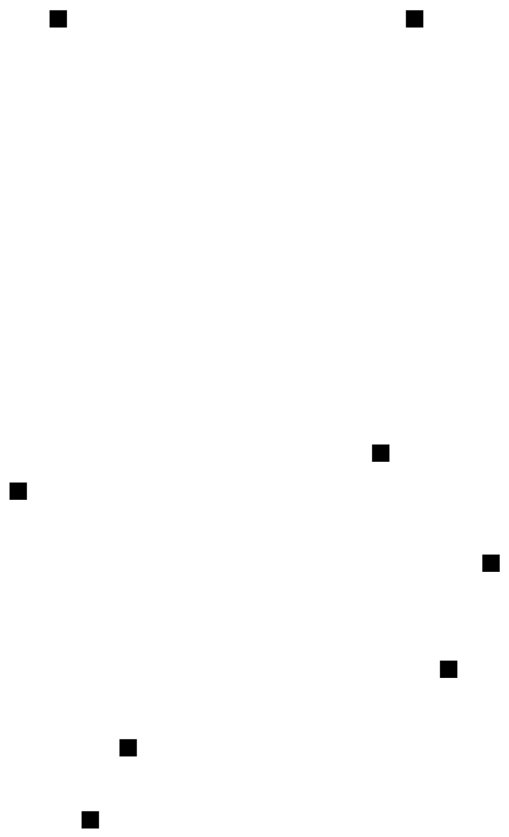

Fig. S10 HMBC spectrum of Ca- glucarate exchange product recovered from H_2O through rotavap drying (50 mbar, 50 ^{o}C). ■ D-Glucaric Acid

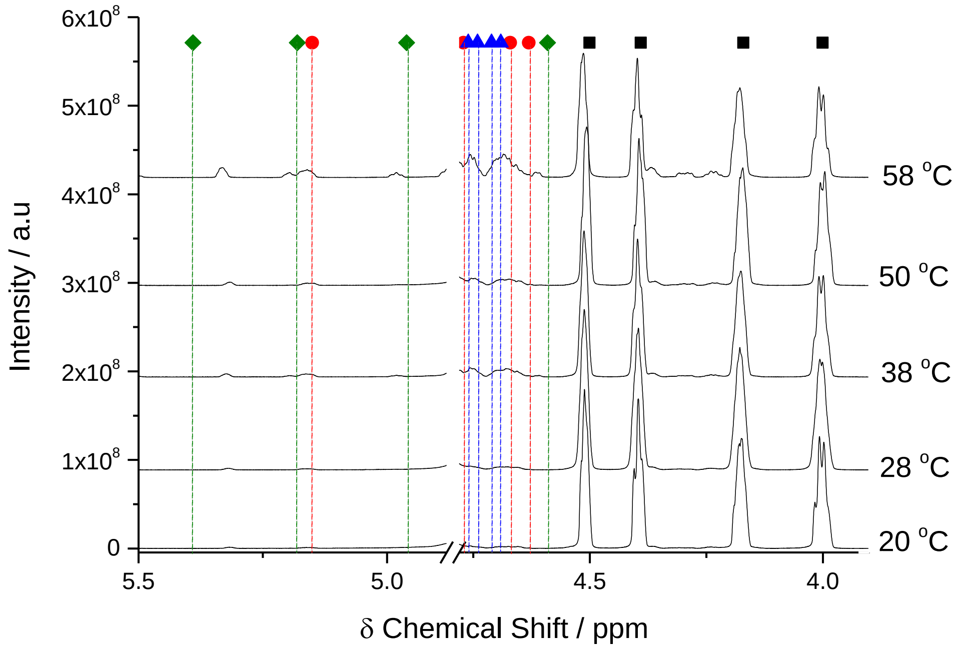

Fig. S11 ^{1}H NMR spectrum of azeotrope- dried Glucaric Acid recovered by evaporation at different temperatures

■ Glucaric Acid$_{ref}$ ● Glucaro 1,4- lactone$_{ref}$, ▲ Glucaro 3,6- lactone$_{ref}$, ◆ Glucaro 1,4 : 3.6 – dilactone$_{ref}$

X-ray crystal structure data for as prepared D-glucaric acid

Identification code	D-glucaric acid	
Empirical formula	C6 H10 O8	
Formula weight	210.14	
Temperature	293(2) K	
Wavelength	1.54184 Å	
Crystal system	Monoclinic	
Space group	P 21	
Unit cell dimensions	a = 6.7810(3) Å	[illegible]= 90°.
	b = 8.6507(3) Å	[illegible]= 93.587(4)°.
	c = 7.2443(3) Å	[illegible] = 90°.
Volume	424.12(3) $Å^3$	
Z	2	
Density (calculated)	1.645 Mg/m^3	
Absorption coefficient	1.404 mm^{-1}	
F(000)	220	
Crystal size	0.379 x 0.165 x 0.119 mm^3	
Theta range for data collection	6.121 to 74.155°.	
Index ranges	-8<=h<=8, -10<=k<=10, -9<=l<=9	
Reflections collected	5989	
Independent reflections	1669 [R(int) = 0.0166]	
Completeness to theta = 67.684°	99.5 %	
Refinement method	Full-matrix least-squares on F^2	
Data / restraints / parameters	1669 / 1 / 133	
Goodness-of-fit on F^2	1.042	
Final R indices [I>2sigma(I)]	R1 = 0.0220, wR2 = 0.0569	
R indices (all data)	R1 = 0.0222, wR2 = 0.0571	
Absolute structure parameter	-0.01(6)	
Extinction coefficient	n/a	
Largest diff. peak and hole	0.180 and -0.127 e.$Å^{-3}$	

Table S1. Atomic coordinates (x 10^4) and equivalent isotropic displacement parameters ($Å^2$x 10^3) for D-glucaric acid. U(eq) is defined as one third of the trace of the orthogonalized U^{ij} tensor.

	x	y	z	U(eq)
C(1)	-2395(2)	9437(2)	5141(2)	27(1)
C(2)	-2830(2)	8368(2)	6734(2)	23(1)
C(3)	-1097(2)	7240(2)	7043(2)	23(1)
C(4)	-1210(2)	6309(2)	8827(2)	24(1)
C(5)	-3102(2)	5352(2)	8958(2)	28(1)
C(6)	-3119(3)	4051(2)	7534(3)	32(1)
O(1)	-2383(2)	10835(2)	5263(2)	39(1)
O(2)	-2023(2)	8673(2)	3643(2)	36(1)
O(3)	-3056(2)	9244(2)	8351(2)	30(1)
O(4)	692(2)	8114(2)	7163(2)	30(1)
O(5)	433(2)	5260(2)	8942(2)	30(1)
O(6)	-3279(2)	4843(2)	10788(2)	34(1)
O(7)	-2921(3)	2662(2)	8262(2)	47(1)
O(8)	-3264(2)	4302(2)	5890(2)	43(1)

Table S2. Bond lengths [Å] and angles [°] for D-glucaric acid.

C(1)-O(1)	1.213(2)
C(1)-O(2)	1.309(2)
C(1)-C(2)	1.522(2)
C(2)-O(3)	1.412(2)
C(2)-C(3)	1.533(2)
C(3)-O(4)	1.427(2)
C(3)-C(4)	1.529(2)
C(4)-O(5)	1.4348(19)
C(4)-C(5)	1.535(2)
C(5)-O(6)	1.409(2)
C(5)-C(6)	1.526(3)
C(6)-O(8)	1.208(2)
C(6)-O(7)	1.316(2)
O(1)-C(1)-O(2)	124.26(18)
O(1)-C(1)-C(2)	123.48(17)
O(2)-C(1)-C(2)	112.26(14)
O(3)-C(2)-C(1)	109.84(14)
O(3)-C(2)-C(3)	110.04(13)
C(1)-C(2)-C(3)	108.30(13)
O(4)-C(3)-C(4)	108.34(13)
O(4)-C(3)-C(2)	108.23(13)
C(4)-C(3)-C(2)	112.34(13)
O(5)-C(4)-C(3)	107.59(12)
O(5)-C(4)-C(5)	107.75(14)
C(3)-C(4)-C(5)	114.99(14)
O(6)-C(5)-C(6)	114.05(15)
O(6)-C(5)-C(4)	110.30(14)
C(6)-C(5)-C(4)	109.09(14)
O(8)-C(6)-O(7)	123.99(19)
O(8)-C(6)-C(5)	122.03(17)
O(7)-C(6)-C(5)	113.97(16)

Symmetry transformations used to generate equivalent atoms:

Table S3. Anisotropic displacement parameters ($Å^2$x 10^3) for D-glucaric acid. The anisotropic displacement factor exponent takes the form: $-2\pi^2[h^2a^{*2}U^{11} + ... + 2 h k a^* b^* U^{12}]$

	U_{11}	U_{22}	U_{33}	U_{23}	U_{13}	U_{12}
C(1)	28(1)	26(1)	27(1)	1(1)	3(1)	-2(1)
C(2)	24(1)	25(1)	22(1)	-1(1)	3(1)	-2(1)
C(3)	24(1)	23(1)	22(1)	-3(1)	4(1)	-3(1)
C(4)	26(1)	25(1)	21(1)	-2(1)	3(1)	3(1)
C(5)	26(1)	29(1)	28(1)	6(1)	4(1)	3(1)
C(6)	29(1)	30(1)	35(1)	5(1)	-2(1)	-6(1)
O(1)	56(1)	26(1)	38(1)	3(1)	14(1)	-4(1)
O(2)	52(1)	32(1)	25(1)	0(1)	10(1)	-5(1)
O(3)	28(1)	34(1)	27(1)	-7(1)	6(1)	3(1)
O(4)	25(1)	36(1)	31(1)	-6(1)	8(1)	-7(1)
O(5)	29(1)	39(1)	23(1)	-2(1)	-2(1)	8(1)
O(6)	31(1)	40(1)	32(1)	12(1)	10(1)	7(1)
O(7)	76(1)	26(1)	39(1)	3(1)	-3(1)	-2(1)
O(8)	58(1)	38(1)	31(1)	4(1)	-7(1)	-12(1)

Table S4. Hydrogen coordinates (x 10^4) and isotropic displacement parameters ($Å^2$x 10^3) for D-glucaric acid.

	x	y	z	U(eq)
H(2)	-4046	7788	6419	28
H(3)	-1083	6529	5990	27
H(4)	-1085	7019	9882	29
H(5)	-4229	6030	8630	33
H(2O)	-1614	9272	2877	54
H(3O)	-4235	9380	8486	44
H(4O)	1414	7816	6368	45
H(5O)	1000	5317	9973	46
H(6O)	-2337	4287	11101	51
H(7O)	-2829	2023	7436	71
